# Persistence and spread of fluconazole-resistant Candida parapsilosis clinical isolates associated with increased ERG11 copies in Qatar

**DOI:** 10.1099/mgen.0.001653

**Published:** 2026-02-20

**Authors:** Zhu Zhang, Yue Wang, Fatma Ben Abid, Husam Salah, Khalil Al Ismail, Haneen Eldos, Bincy Samuel, Ruwen Zhou, Sathyavathi Sundararaju, Mohammed Suleimen, Kun Wang, Bernett Lee, Muna Al Maslamani, Ali A. Sultan, Andres Perez-Lopez, Jianping Xu, Clement K.M. Tsui

**Affiliations:** 1 Lee Kong Chian School of Medicine, Nanyang Technological University, Singapore, Singapore; 2 Department of Biology, McMaster University, Hamilton, Ontario, Canada; 3 Department of Medicine, Division of Infectious Diseases, Hamad Medical Corporation, Doha, Qatar; 4 Weill Cornell Medicine-Qatar, Doha, Qatar; 5 Communicable Disease Centre, Hamad Medical Corporation, Doha, Qatar; 6 College of Medicine, Qatar University, Doha, Qatar; 7 Division of Microbiology, Department of Laboratory Medicine and Pathology, Hamad Medical Corporation, Doha, Qatar; 8 Department of Microbiology and Immunology, Weill Cornell Medicine-Qatar, Doha, Qatar; 9 Division of Microbiology, Department of Pathology, Sidra Medicine, Doha, Qatar; 10 Department of Biomedical Sciences, Qatar University, Doha, Qatar; 11 Research Department, Sidra Medicine, Doha, Qatar; 12 Infectious Diseases Research Laboratory, National Center for Infectious Diseases, Singapore, Singapore; 13 Faculty of Medicine, University of British Columbia, Vancouver, BC, Canada

**Keywords:** azole resistance, candidemia, copy number variation, gene expression, nosocomial, molecular, Middle East

## Abstract

*Candida parapsilosis* is one of the most common species associated with candidemia infections globally. Recently, the emergence of fluconazole-resistant (FLU-R) *C. parapsilosis* has become a significant global public health concern. In this study, we investigated the genomic epidemiology and potential mechanisms of antifungal resistance among 51 hospital isolates in Qatar, of which 18 were FLU-R. Whole-genome SNP analysis revealed the presence of five major genetic clusters and evidence for cross-hospital transmission within clusters I, II and III. Cluster I had 21 isolates, including all 18 FLU-R isolates collected from 2015 to 2021. These 18 FLU-R isolates had no missense variants known to be associated with azole resistance in loci such as *ERG11, ERG6* and *TAC1*; however, all FLU-R isolates had increased copy numbers of *ERG11*, ranging from 3 to 7 copies. In addition, most FLU-R isolates (*n*=16) had increased *CDR1B* copies (2–8 copies). These FLU-R isolates also had higher expression of *ERG11* and *CDR1B* than sensitive strains. A genome-wide association study revealed 16 variants in several loci of unknown function that may be linked to resistance to FLU and 5-flucytosine. All cluster I isolates had unique missense mutations in *EFG1* and *UME6* that may play important roles in morphogenesis and biofilm formation. Our findings indicate these cluster I isolates may have evolved a greater propensity to persist within hospitals for prolonged periods and cause clonal transmission than isolates in other genetic clusters susceptible to fluconazole.

**Impact Statement**

*Candida parapsilosis* is a WHO priority fungal pathogen, accounting for an increasing proportion of invasive *Candida* infection worldwide. Many of these invasive infections are caused by azole-resistant strains in clinical settings. However, its genomic diversity, antifungal resistance mechanisms and dynamics remain poorly understood globally. In this work, we investigated the genetic relationships and molecular mechanisms associated with 51 Qatari *C. parapsilosis* isolates using the whole-genome sequencing approach. Our data revealed the presence of five major genetic clusters and identified the persistence and transmission of fluconazole-resistant (FLU-R) *C. parapsilosis* isolates in various hospitals. Yet, these FLU-R isolates did not have the Y132F mutation in the Erg11 protein, which is commonly associated with FLU-R. Instead, all FLU-R *C. parapsilosis* isolates had increased copy number of *ERG11*, and most FLU-R isolates had increased *CDR1B* copies, indicating that copy number variation plays an important role in conferring fluconazole resistance via increased gene expression. Our data highlights the need to understand the epidemiology of invasive *C. parapsilosis* and antifungal resistance mechanisms and to inform infection control strategy.

## Data Availability Statement

The raw sequencing reads are available from the National Center for Biotechnology Information (NCBI) under the BioProject accession number PRJNA997443. BioSample and SRA accession numbers are listed in Table S1. Codes for CNV analysis are available in https://github.com/tequilasunrise0/Candida-CNV-analysis.

## Introduction

*Candida parapsilosis* is a common cause of infections in humans and is reported to be the second to fourth most prevalent *Candida* species causing invasive candidemia in various geographical regions [1,2]. For instance, *C. parapsilosis* is the second major cause of candidemia in Japan [3], Spain [4,5] and Iran [6]. Since the early 2000s, the clinical prominence and incidence of *C. parapsilosis* and other non-*albicans Candida* species have increased significantly and have become an emerging public health concern [7]. It colonizes the human skin and mucosal membranes as a commensal micro-organism, wherein the hands of healthcare professionals are recognized as a major vector for *C. parapsilosis* nosocomial acquisition [2]. Therefore, immunocompromised individuals including patients with human immunodeficiency virus-1 infection and cancer, neonates with prolonged stays at neonatal intensive care units (ICUs) and surgical patients, especially those undergoing gastrointestinal procedures, are at high risk for *C. parapsilosis* infection [7,8]. In 2022, *C. parapsilosis* was listed as a ‘High Priority’ fungal pathogen by the WHO [9].

*C. parapsilosis* is diploid with an estimated haploid genome size of 13.1 Mb. Compared with *Candida albicans* and *Candida tropicalis*, it has a relatively low level of heterozygosity with an average of one SNP per 15,553 bases within individual strains [8,10, 11]. *C. parapsilosis* has not been reported to mate and is assumed to reproduce predominantly asexually due to its pseudogenization of the *MTL***a** locus and the lack of *MAT***α** idiomorph among the analysed strains [11,12].

Although this species had been considered susceptible to most antifungals, recent reports have indicated increasing resistance to fluconazole in *C. parapsilosis* globally, particularly in isolates associated with invasive infections [1,6, 13]. Although resistance rates vary broadly by country and region, some studies have reported fluconazole-resistant (FLU-R) *C. parapsilosis* rates as high as 80% [5,14, 15]. This problem is compounded by the reduced susceptibility of this species to echinocandins with increased minimum inhibitory concentration (MIC) to these agents [3,16]. Azole resistance in *Candida* species may arise from changes to the target gene *ERG11* that codes for a key enzyme in the ergosterol biosynthesis pathway targeted by azoles, or gain-of-function mutations in transcription factors *MRR1* and *TAC1*, leading to the up-regulation of the major facilitator efflux superfamily (Mdr1) or ATP-binding cassette transporter (Cdr1 and Cdr2), which can act as azole efflux pumps [1,10, 17]. In *C. parapsilosis*, aside from mutations in the above genes, mutations in other genes such as *ERG3* and *ERG6* have also been linked to azole resistance [17,24]. Indeed, nosocomial outbreaks caused by *C. parapsilosis,* particularly FLU-R clones, have emerged in many countries such as South Korea, Brazil, Turkey, France, India, Germany, Austria, China, the UK and Italy [1,5, 16, 22,31]. Among these investigations, isolates/clones harbouring mutations in the Erg11 enzyme such as the Y132F mutation were prevalent; 30 and 50% FLU-R isolates carrying the Erg11 Y132F mutation were reported in South Korea and China, respectively [24,27].

Patients with severe coronavirus disease 2019 (COVID-19) are known to be susceptible to acquiring secondary fungal infections such as candidemia, and outbreaks of COVID-19-associated candidemia due to *Candidozyma auris* (formerly *Candida auris*), *Nakaseomyces glabratus*, *C. parapsilosis* and *C. tropicalis* have been reported in many countries, including Qatar [18,32,37]. The application of whole-genome sequencing (WGS) has allowed for a more detailed understanding of population structure, evolution and geographic dissemination of these *Candida* pathogens. However, the biology and epidemiology of *C. parapsilosis* have not been as extensively explored as those of *C. auris* and *N. glabratus*. Because *C. parapsilosis* poses an increasing clinical challenge in the management of fungal infections in vulnerable patient populations, understanding the origin, transmission, genetic relationships among isolates and molecular mechanisms underlying their drug resistance will aid in the development of new diagnostic tools and effective antifungal therapies. In this investigation, we used the WGS approach to study the genetic relationships of the Qatari *C. parapsilosis* isolates and to determine the possible pattern of transmission, particularly those associated with COVID-19 patients. Also, we characterized the molecular mechanisms, such as variants and copy number variation (CNV), as well as the expression of selected genes, that could be associated with differences in susceptibility to fluconazole. In addition, we studied the genomic relatedness between Qatari isolates and those from other parts of the world.

## Methods

### Sample collection, identification and antifungal susceptibility testing

This study was based on the retrospective collection of *C. parapsilosis* stored in Hamad Medical Corporation and Sidra Medicine (SM) between January 2015 and December 2022. The collection includes strains that were isolated from patients from six different hospitals in Qatar, including Hamad General Hospital (HGH), Hazm Mebaireek Hospital (HMGH), Heart Hospital (HH), Rhumailah Long Term Facility (RH), SM and National Center for Cancer Care and Research (NCCCR) between 2015 and 2022. During the COVID-19 pandemic, HMGH was one of the facilities dedicated to caring for patients with severe lower respiratory tract infections, particularly those who required critical care. Isolates were recovered mainly from blood and sterile body fluids like pleural fluid and the cerebrospinal fluid (CSF). Isolates were cultured on Chromogenic *Candida* agar (CHROMagar, France) at 37 °C for 5–7 days following the manufacturer’s recommendations. Species identities were confirmed using MALDI-TOF MS (Bruker Daltonics, Bremen, Germany). The isolates were maintained on Sabouraud dextrose agar at 4 °C before DNA extraction.

MICs of each isolate for amphotericin B (Amb), fluconazole (Flu), itraconazole (Ita), voriconazole (Vor), posaconazole (Pos), flucytosine (Fc), anidulafungin (And), caspofungin (Casp) and micafungin (Mica) were measured using either Sensititre™ YeastOne (TREK Diagnostic Systems, Cleveland, OH, USA), Vitek2 (bioMérieux, Inc., Hazelwood, MO) or Etest (bioMérieux, Inc.) at various hospital sites. Clinical and Laboratory Standards Institutes (CLSI) breakpoints were used to interpret MICs for Flu, And, Casp and Mica [38]. In addition, CLSI epidemiological cutoff values (ECVs) were used for Amb, Ita and Pos to separate WT from non-WT isolates. There is no CLSI breakpoint or ECV for Fc. Quality control strains, *C. parapsilosis* ATCC 22019 and *C. albicans* ATCC 90028, were tested using the same antifungal susceptibility testing methods and MICs interpreted following the CLSI guidelines (CLSI M27M44S).

Epidemiological data such as date of patient admission were reviewed to determine if these isolates were from hospital-acquired infection (HAI) or community-acquired infection based on the Qatar National Healthcare Safety Network (NHSN) definition. HAI was defined if the date of the event of the NHSN site-specific infection criterion occurred on or after the third calendar day of admission to an inpatient location where the day of admission was calendar day 1. If the infection was identified within 2 days before admission or the day of admission to an inpatient location (calendar day 1) or the calendar day 2 after admission, the infection was considered present on admission.

### Whole-genome sequencing

Genomic DNA was extracted from each isolate using MasterPure Yeast DNA purification kit (Lucigen Corporation, WI, USA) and quantified using Qubit 2 fluorometer (ThermoFisher, MA, USA). Genomic DNA libraries were constructed with Nextera XT library preparation kit or DNA preparation kit (Illumina Inc., USA), and the libraries were sequenced on Illumina NextSeq for 300 cycles (150 bp paired-end) at the Integrated Genomics Services Department, SM.

### Mapping, variant callings and bioinformatics analysis

Raw read qualities were assessed by Fastqc v0.11.9 (https://www.bioinformatics.babraham.ac.uk/projects/fastqc/). The sequence reads were trimmed by Trimmomatic v0.39 [39] to remove the adapters and low-quality reads. To determine the genetic relationships among isolates, we analysed the SNPs using two approaches. Firstly, the trimmed and filtered reads of each strain were mapped to the reference genome CDC317 (ASM18276v2) of *C. parapsilosis* using BWA v0.7.17 [40]. Alignments were then converted to BAM format and sorted by chromosomes using SAMtools v1.13 [41,42]. Meanwhile, duplicated reads were labelled using Picard v2.26.3 (https://broadinstitute.github.io/picard/). Next, genomic variants for individual strains were identified using GATK v4.2.5 HaplotypeCaller in GVCF mode [43]. GVCF files were merged and genotyped using CombineGVCFs and GenotypeGVCFs, respectively. Afterwards, SNPs and INDELS were extracted from the merged GVCF file using SelectVariants and filtered using VariantFiltration with the following hard filters, QD <2.0, FS >60.0, SOR >3.0, MQ <40.0, MQRankSum <−12.5 and ReadPosRankSum <−8.0. Annotation was conducted using SnpEff v5.0 [44]. Variants from a panel of 53 genes previously known to be associated with antifungal resistance and stress adaptations (Table S5, available in the online Supplementary Material) were screened from each VCF file [2,8, 45, 46].

To construct the phylogenetic tree, SNP loci were concatenated into sequences using vcf2phylip v2.0 (https://github.com/edgardomortiz/vcf2phylip), with CDC317 as the reference. IQ-TREE [47] was used to perform the phylogenetic analysis among isolates based on the concatenated SNPs data and to help define phylogenetic clusters. The phylogenetic tree was visualized using iTOL [48]. Pairwise genetic distances between isolates were calculated based on the SNP alignment as well as analysed using Wilcoxon rank-sum tests [49] (stats package, R v4.2.0) across three comparisons: COVID-19 vs. non-COVID-19 samples, cluster I vs. non-cluster I samples and COVID-19 vs. non-COVID-19 samples within cluster I. Benjamini–Hochberg correction was applied for multiple testing, and effect sizes were computed using the effsize package in R [50].

To determine and confirm the mating type of the isolates for all *C. parapsilosis* isolates, quality trimmed reads were assembled using SPAdes v3.15.5 [51] and then assessed using Quast v5.0.2 [52]. The presence or absence of *MAT*α in our isolates was screened using blastn search against the reference genome assembly.

To assess the potential genetic relatedness of the Qatari isolates to the global collections, we compared the Qatari genomes with 228 publicly available *C. parapsilosis* genomes from various countries [23,53], including China (74 isolates), USA (128 isolates), Turkey (7 isolates), Germany (3 isolates), S. Korea (3 isolates) and various other countries.

### Genome-wide association study

In order to uncover novel mutations/variants associated with antifungal resistance in our Qatari population of *C. parapsilosis*, genome-wide association study (GWAS) analyses were conducted to assess the associations between drug susceptibility against nine antifungal drugs and SNPs using both Fixed and random model Circulating Probability Unification (FarmCPU) in GAPIT [54] and Bayesian-information and Linkage-disequilibrium Iteratively Nested Keyway (BLINK) [55]. The nine antifungal drugs were voriconazole (Vor), posaconazole (Pos), itraconazole (Ita), fluconazole (Flu), flucytosine (Fc), caspofungin (Casp), amphotericin B (Amb), anidulafungin (And) and micafungin (Mica).

FarmCPU and BLINK were chosen here because these methods were very efficient and can effectively control for population structure and relatedness among individuals. Specifically, FarmCPU uses a fixed effect model and a random effect model (REM). The fixed effect model is used to assess individual markers while accounting for the influence of pseudo-quantitative trait nucleotides (QTNs), which are significant markers identified in previous iterations. QTNs are evaluated and adjusted for confounding factors through the REM. The process iteratively refines the list of associated markers to identify the most robust associations. Instead of using REM for population structure correction, BLINK uses a Bayesian statistical framework to select the best model and avoid overfitting. Additionally, BLINK uses linkage disequilibrium (LD) information to select a set of pseudo-QTNs that are not in LD, allowing BLINK to focus on the most informative SNPs [55].

### CNV estimation

CNV was characterized for all isolates to investigate its potential association with antifungal resistance, focusing particularly on genes associated with azole resistance. Two approaches were utilized to estimate CNV. Firstly, sequencing depth was assessed using the SAMtools depth module, based on the alignment data in SAM files [41,56]. The CNV for each gene was calculated as the average sequencing depth across the corresponding genomic segment within each gene divided by the average depth of the corresponding chromosome/scaffold. Additional CNV regions across the contigs were predicted using a sliding window approach, where each window spanned 500 bp with no overlap between adjacent windows. The boundaries of CNVs were confirmed by visual inspection in the Integrative Genome Viewer [57]. The association between CNVs of selected loci and MICs was assessed by Kruskal–Wallis test using R 4.0.0.

In addition, CNVpytor v1.3.1 was used to call regions with CNVs [58]. By analysing the sorted.bam files, CNVpytor called regions with CNVs using a read-depth-based approach. The bin size was set to 100 to 1,000 bp. CNV results have been merged over the 51 samples and low-quality calls were filtered. The following filter was used to select reliable CNVs: calls with more than half not uniquely mapped reads, non-confident calls (*P≥*0.0001), calls with more than 50% Ns in the reference genome, calls smaller than 20,000 bp and calls close to gaps in the reference genome (<50,000 bp) were all excluded.

### Genetic diversity and loss of heterozygosity analysis

Genome-wide nucleotide diversity (π) and Tajima’s D (TD) were estimated from 10 kb windows across the genome using jydu/vcftools haploid mode [59] to compare the cluster I and non-cluster I isolates.

In general, heterozygosity in diploid fungi such as *C. parapsilosis* could be lost during both sexual and asexual reproduction. During sexual reproduction, meiotic recombination followed by mating among close relatives would cause reduction in heterozygosity. During asexual reproduction, mitotic recombination between homologous chromosomes or gene conversion would also lead to loss of heterozygosity. However, since only one single mating type has been reported in diploid *C. parapsilosis* among natural isolates, loss of heterozygosity through sexual reproduction is unlikely.

To illustrate the heterozygosity pattern, scaffolds were divided into non-overlapping 5,000 bp windows. And the number of heterozygous SNP loci was calculated in each window and visualized using different intensities of blue colour. The heatmap was generated using Python v3.11.4 matplotlib v3.7.1 [60].

### Expression of *ERG11* and *CDR1b* between resistant and sensitive isolates

We performed quantitative polymerase chain reaction (qPCR) to assess the gene expression of *ERG11* and *CDR1b* between FLU-R and FLU-S *C. parapsilosis* isolates. Briefly, *C. parapsilosis* samples were inoculated into 5 ml of yeast peptone dextrose broth and incubated at 30 °C at 150 r.p.m. Total RNA was extracted from the cell pellets using Quick-RNA Fungal/Bacterial Miniprep kit (Zymo), following the manufacturer’s instructions. An in-column DNase 1 treatment was included to remove residual genomic DNA. cDNA was synthesized from 2 µg of total RNA using High-Capacity cDNA Reverse Transcription kit (ThermoFisher), following the manufacturer’s protocol. The relative expression levels of *ERG11* and *CDR1b* were quantified using SYBR Green-based qPCR through the comparative ΔΔCt method, with ACT1 as the reference. Gene-specific primers for the target genes and the reference gene were listed in the supplementary text. All statistical analyses and visualizations, including boxplots with significance markers, were generated using R v4.4.2.

## Results

### Epidemiology, antifungal susceptibilities and phylogenetic structure

**Fig. 1.**
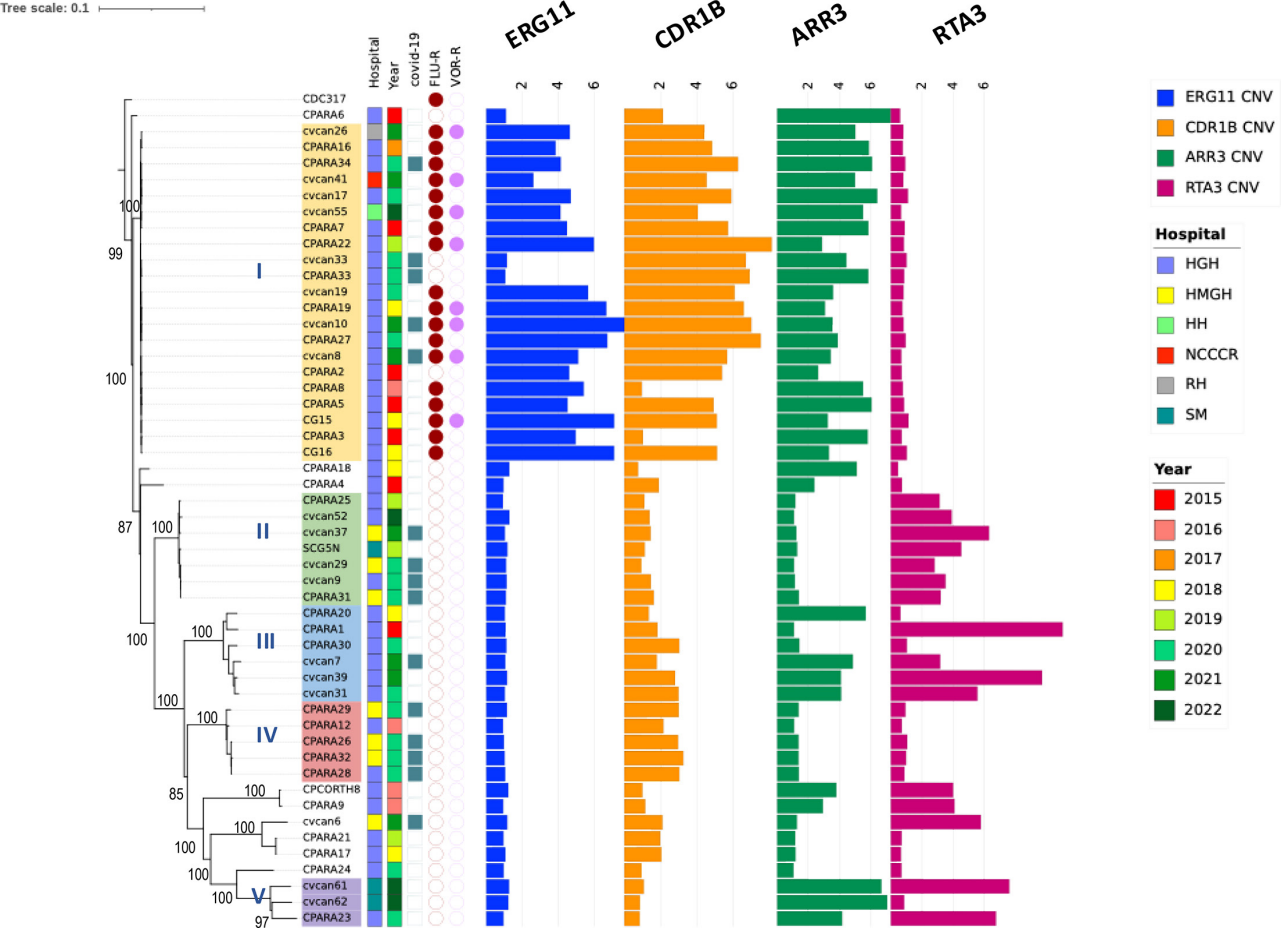
Genetic relationships among Qatari *C. parapsilosis* isolates inferred from whole-genome SNPs using CDC317 as the reference genome. IQ-TREE was performed on an alignment of 25,371 SNPs. The bootstrap values were indicated, and five major genetic clusters (I, II, III, IV and V) are marked with different colours. The tree was overlaid with years of isolation, hospital, countries, COVID-19 status and fluconazole and voriconazole resistance, as well the estimated copy number of four loci: *ERG11, CDR1B, ARR3* and *RTA3* (in which, over ten isolates had increased copies).

A total of 51 *C*. *parapsilosis* strains between 2015 and 2022 were studied. Forty-six were isolated from blood and five from other body fluids such as CSF (Table S1). Fifteen strains were collected from COVID-19 patients during the pandemic between 2020 and 2022. Detailed information on all isolates is summarized in Table S1. A total of 25,371 genome-wide SNPs (0.2% at the genome level) were identified in the dataset, including heterozygous sites within individual strains represented by the IUPAC degenerate nucleotide codes. Overall, most isolates can be divided into 5 major clusters, with 21 belonging to cluster I, 7 to cluster II, 6 to cluster III, 5 to cluster IV and 3 in cluster V, while the remaining 9 isolates were quite distinct from each other and from the 5 clusters (Fig. 1). Based on the homozygous SNPs, the variations within clusters I, II and IV were smaller than those in clusters III and V, and the average number of SNP difference between isolates within individual clusters was 38.1, 77.4, 369.4, 118.1 and 936.3, respectively, for clusters I, II, III, IV and V (Tables 1 and S2). Since a relatively large number of isolates (41%, 21/51) belonged to cluster 1, we further compared the genetic diversity between cluster I and non-cluster I where Tajima’s D (TD) for cluster I and non-cluster I were −0.94 vs. −0.86 (−1.05 for all isolates) (Table S3). The negative values in both groups are consistent with recent clonal population expansion.

**Table 1.** Average homozygous SNP difference (and SNP difference in range) among *C. parapsilosis* isolates within and between the five phylogenetic clusters

Cluster	I	II	III	IV	V
I	**38.1** (20–64)	981.2 (959–1007)	1,881.7 (1,819–1,938)	1,913 (1,893–1,943)	3,054.9 (2,999–3,152)
II		**77.4** (20–125)	1,845.5 (1,777–1,903)	1,861.4 (1,840–1,894)	3,040.4 (2,977–3,148)
III			**369.4** (121–520)	1,588.2 (1,529–1,640)	2,878.1 (2,785–3,008)
IV				**118.1** (43–153)	2,780.4 (2,709–2,886)
V					**936.3** (901–981)

There was no obvious pattern of temporal clustering or clustering based on country of origins for patients, as isolates from patients originated in different countries and years were broadly distributed across the phylogenetic tree (Fig. 1). The molecular epidemiological data suggest the following. First, clones and clonal clusters persisted within hospitals. Specifically, cluster I isolates were detected in HGH between 2015 and 2021, and isolates belonging to different genetic clusters were detected in HGH as well. Second, closely related isolates within several clonal clusters were shared among hospitals and among unrelated individuals. For example, closely related isolates of both clusters II and IV were found in two hospitals, HGH and HMGH, and from both unrelated COVID-19 and non-COVID-19 patients. In addition, 15 COVID-19-related isolates did not concentrate in one cluster but were distributed in three main clusters (I, II and VI), while one isolate (cvcan6) was genetically different from those in the three clusters, and even though most isolates were from HGH and HMGH (Fig. 1).

The SNP data revealed evidence for nosocomial transmission of *C. parapsilosis* within and between the hospitals in Qatar. Among others, there were several clusters of COVID-19-associated isolates (Fig. S1a, b), particularly within cluster I (Fig. S1c). For example, the average SNP difference (including heterozygous sites) among COVID-19 isolates in cluster I was 546.6 (350–670), while the difference among non-COVID-19 isolates was 1,553.7 (367–3,087) (Table S2). Except for isolates CPARA12 and CPARA20, all other *C. parapsilosis* isolates were considered HAI. Many patients were transferred between wards, ICUs and hospitals. After reviewing the patient epidemiological information, particularly during the COVID-19 pandemics (2020–2021), we identified several putative hospital transmission events/chains among isolates in clusters I, III and IV within HGH and HMGH (Table S4).

Antifungal susceptibility data were available for all 51 isolates (Table S1). Eighteen (35%) isolates were resistant to fluconazole, 5 (10%) isolates were fluconazole susceptible dose-dependent (SDD) and the remaining 28 were susceptible to fluconazole. All 51 isolates were susceptible to echinocandins. In addition, 8 FLU-R isolates were cross-resistant to voriconazole (MIC ≥1 µg ml^−1^). With respect to antifungal drugs without CLSI breakpoints, such as Amb, Ita and Pos, we found 0, 8 and 15 isolates with their MICs greater than or equal to their ECVs. The remaining samples were sensitive to all antifungal drugs.

All 18 FLU-R isolates between 2015 and 2021 belonged to cluster I, and the prevalence of FLU-R within this cluster was high (85.7%, 18 out of 21). In addition, two other isolates (cvcan33 and CPARA33) in this cluster were SDD (4 µg ml^−1^), while isolate CPARA2 had low MICs (2 µg ml^−1^). All the non-cluster I isolates were susceptible to fluconazole, except two SDD isolates (CPARA17 and CPARA21). Among the 18 patients infected by FLU-R isolates, 15 were in the ICU, 14 received echinocandin treatment (And or Casp) and 4 received Flu. Among patients with FLU-R isolates, 9 had microbiological cure, while 12 did not survive.

To investigate the genetic relationships between Qatari isolates and the global collections, we retrieved the raw sequence reads from 228 global isolates in the NCBI. The phylogenetic tree demonstrated that the Qatari isolates were diverse and intermixed with isolates from other countries. Four of the five major clusters (except cluster V) had no closer relatives from other parts of the world than among themselves, suggesting that this cluster might be unique to Qatar/Middle East (Fig. S2).

### Mechanisms of azole resistance and adaptation

To explore the possible mechanism of fluconazole resistance, we looked for SNPs and structural variants in a panel of genes/loci (Table S5) that had previously shown to be associated with antifungal resistance and adaptations [2,8]. Those included genes coding for lanosterol 14-*α*-demethylase (Erg11), a major facilitator superfamily transporter (Mdr1), ABC transporters (Cdr1, Cdr2) and transcription factors (Mrr1, Tac1) thought to control the transcription of *ERG11* (CPAR2_303740), *CDR1* (CPAR2_405290), *MDR1* (CPAR2_301760), *CDR2* (CPAR2_401370) and *MDR1B* (CPAR2_603010) genes [2]. However, there were no missense variants and stop codons in these loci that demonstrated apparent association with reduced fluconazole susceptibilities (Fig. S3, Table S6). All isolates carried the WT amino acid at 132Y of Erg11, whose missense mutation 132F has been associated with frequent fluconazole resistance in *C. parapsilosis*. However, the R398I mutation at Erg11 was detected in six fluconazole-sensitive isolates (cvcan7, cvcan31, cvcan39, CPARA30, CPARA1 and CPARA20). Other common mutations in Erg11 such as K143R reported in several studies were not detected among these Qatari isolates. None of the isolates carried any mutations in known hotspot regions of *FKS1* (CPAR2_106400), consistent with their sensitivity to echinocandins.

Apart from antifungal resistance, we also detected mutations in candidate loci that may be associated with host infection and adaptations in specific cluster [2,8, 46] (Fig. S3, Table S5). For example, we detected a missense mutation (I280T) in Efg1 (CPAR2_701620) in all cluster I isolates. Mutation (T46K) in Ume6 (CPAR2_803820) was also detected in 21 cluster I isolates, including both FLU-S and FLU-R strains (Fig. S3). *EFG1* and *UME6* play important roles in morphogenesis and biofilm formation in *C. parapsilosis* [61,62].

Since no previously published missense and nonsense mutations/variants were detected in loci known to be associated with antifungal resistance [such as *ERG3* (CPAR2_105550), *ERG6* (CPAR2_405010) or *ERG11* and *MDR1*] in our Qatari population, we performed GWAS to identify potential novel SNPs associated with variations in nine antifungal susceptibilities. Given the small sample size in this study, the findings were interpreted with caution. GWAS analyses identified 16 SNPs associated with reduced susceptibility to two (FLU and Fc) of the nine drugs (Tables 2 and S7). Out of 14 putative SNPs/indels associated with FLU-R, a stop codon (Gly1649*) was detected in the product of CPAR2_806430, which is known to be involved in mitotic division septum assembly. Also, a missense mutation was identified in CPAR2_203680, a gene known to play a role in actin cortical patch organization, endocytosis and endoplasmic reticulum unfolded protein response. Similarly, a missense mutation was found in CPAR2_102380 that has predicted nucleic acid binding activity (Fig. S4). In addition, one synonymous mutation (c.1470G>A, p.Ser490Ser) was detected in Als3 (CPAR2_404770), a putative adhesin (Fig. S4). Ten additional mutations were detected in the upstream of seven different ORFs (Tables 2 and S7). For Fc, a missense mutation (p.Ile74Val) was found to be significantly associated with susceptibility differences in Als*3* (CPAR2_404770), the putative adhesin-encoding gene.

**Table 2.** Details of SNPs identified through GWAS

Drug	SNP position	*P* value	Variant type	HGVS.c	HGVS.p	Gene_id	Gene	Annotation (from Candida Genome Database)
**Flu**	SNW_023503277.1_30338	4.10E−28	Upstream	c.-3357C>A		CPAR2_600100		Orthologues have mRNA binding activity, role in telomere maintenance and cytoplasm, nucleus localization
	SNW_023503278.1_1560502	6.02E−29	Synonymous	c.327C>T	p.Ile109Ile	CPAR2_107350		
	SNW_023503278.1_550010	8.18E−22	Missense	c.227A>G	p.Gln76Arg	CPAR2_102380		Has domain(s) with predicted nucleic acid binding activity
	SNW_023503280.1_1483690	6.91E−09	Stop_gained	c.4945G>T	p.Gly1649*	CPAR2_806430		Orthologues have role in mitotic division septum assembly, protein localization to bud neck and cell division site, cytoplasm, division septum localization
	SNW_023503281.1_1627857	6.91E−09	Upstream	c.-340T>G		CPAR2_207600		
	SNW_023503281.1_1698421	2.55E−08	Upstream	c.-4837G>T		CPAR2_207880		Orthologues have protein tag activity and role in mitotic recombination-dependent replication fork processing, protein sumoylation, stalled replication fork localization to nuclear periphery, telomere maintenance
	SNW_023503281.1_1698432	2.55E−08	Upstream	c.-4848C>A		CPAR2_207880		Orthologues have protein tag activity and role in mitotic recombination-dependent replication fork processing, protein sumoylation, stalled replication fork localization to nuclear periphery, telomere maintenance
	SNW_023503281.1_2374745	6.91E−09	Upstream	c.-92A>C		CPAR2_211150		Orthologues have RNA polymerase I activity and role in nucleolar large rRNA transcription by RNA polymerase I, transcription by RNA polymerase I, transcription elongation by RNA polymerase I
	SNW_023503281.1_2374752	6.91E−09	Upstream	c.-99A>C		CPAR2_211150		Orthologues have RNA polymerase I activity and role in nucleolar large rRNA transcription by RNA polymerase I, transcription by RNA polymerase I, transcription elongation by RNA polymerase I
	SNW_023503281.1_2637591	3.10E−30	Upstream	c.-247A>T		CPAR2_212210	ALD5	Putative aldehyde dehydrogenase
	SNW_023503281.1_748745	6.91E−09	Missense	c.2065A>C	p.Ser689Arg	CPAR2_203680		Orthologues have ubiquitin binding activity and role in actin cortical patch organization, endocytosis, endoplasmic reticulum unfolded protein response, positive regulation of cytokinesis, regulation of protein localization
	SNW_023503283.1_1070076	5.50E−08	Synonymous	c.1470G>A	p.Ser490Ser	CPAR2_404770	ALS3	Putative adhesin
	SNW_023503283.1_511022	2.35E−08	Upstream	c.-4448A>C		CPAR2_402290		Orthologues have gamma-glutamylcyclotransferase activity and role in glutathione catabolic process
	SNW_023503284.1_252223	2.00E−15	Upstream	c.-4222G>A		CPAR2_701130		Has domain(s) with predicted phospholipase activity and role in phospholipid catabolic process
**Fc**	SNW_023503283.1_1068884	2.95E−27	Missense	c.278T>A	p.Val93Asp	CPAR2_404770	ALS3	Putative adhesin
	SNW_023503283.1_342413	5.26E−15	Synonymous	c.861G>T	p.Gly287Gly	CPAR2_401550		Has domain(s) with predicted hydrolase activity, metallocarboxypeptidase activity and role in nitrogen compound metabolic process

**Fig. 2.**
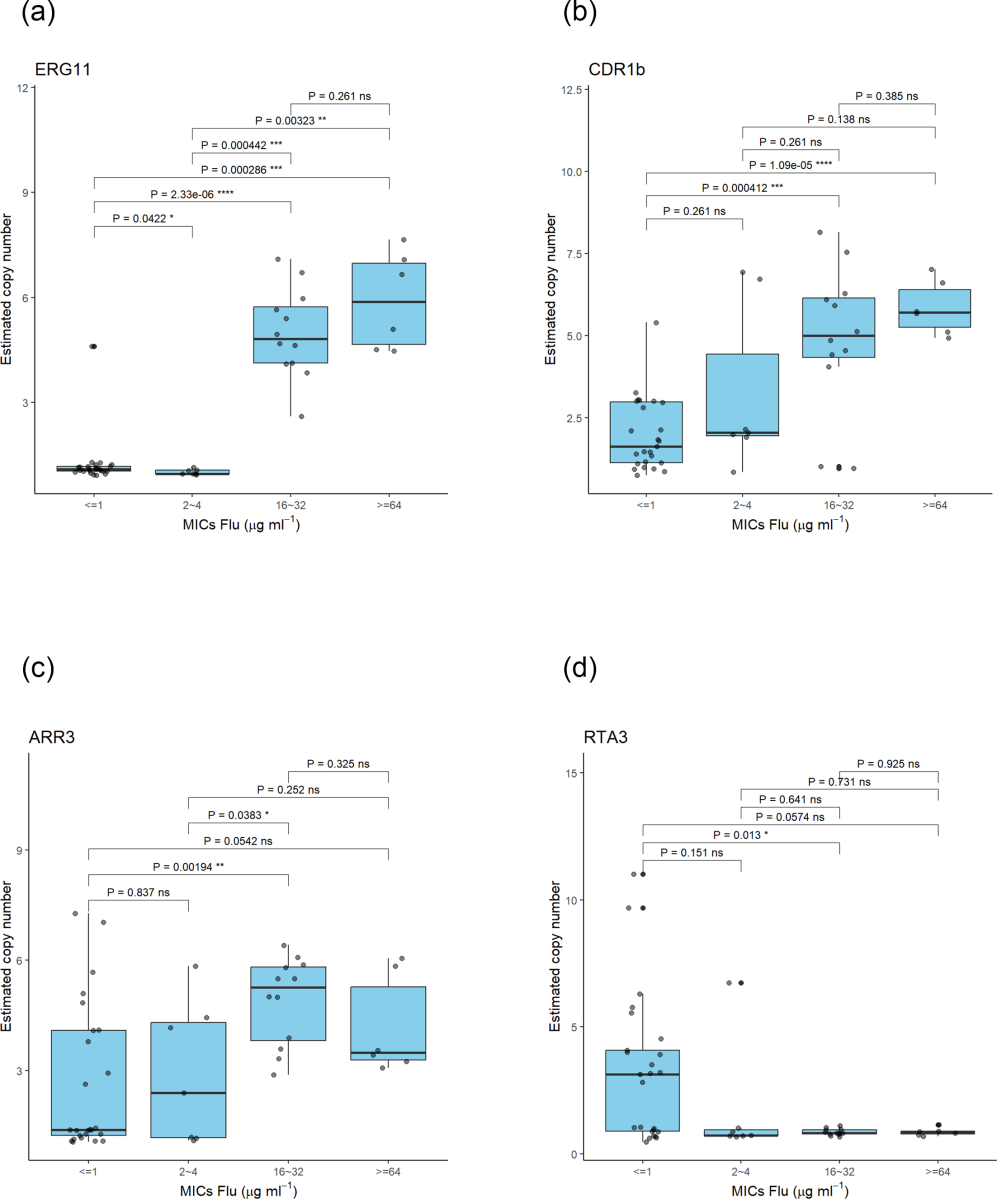
Correlation between estimated copy numbers of four loci and fluconazole (Flu) MICs in Qatari *C. parapsilosis* isolates. (**a**) *ERG11* (*P*<0.000001), (**b**) *CDR1B* (*P*=0.0002), (**c**) *ARR3* (*P*=0.011) and (**d**) *RTA3* (*P*=0.09)*.* The association was assessed by Kruskal–Wallis test using R 4.0.0.

CNVs are important mechanisms of genomic and phenotypic variations and can provide rapid adaptation to stressful environments and conditions [63]. In recent years, CNV has been reported as associated with FLU-R in *C. albicans*, *C. parapsilosis* and *C. tropicalis* [64,67]. Therefore, we performed CNV analysis for the panel loci known to be associated with FLU-R in the genomes of all 51 isolates. First, the ‘samtools depth’ module estimation approach indicated that all FLU-R isolates of cluster I carried more than two copies of *ERG11* (CNV >2) per haploid genome (Fig. 1), while sensitive isolates carried a normal copy number (CNV=1 or below) of *ERG11* per haploid genome (Fig. 1). Using Integrated Genomic Viewer (IGV), the entire ORFs of *ERG11*, as well as the upstream gene *THR1* and downstream gene *HMS1* (2,814 bp; 868,152–870,965 bp), showed increased copy numbers among all the isolates having elevated *ERG11* copies (Fig. S5). All the isolates had the same CNV breakpoints. Apart from *ERG11*, CNV differences were also detected in *CDR1B*, *ARR3*, *RTA3*, *GZF3*, *ERG28*, *LIP1* and *LIP2* (Fig. 1, Table S8). More importantly, an increased *CDR1B* copy number (CNV=2–8) was detected in 30 out of 51 isolates, including all cluster I isolates (Fig. 1). Among the 30 isolates with elevated *CDR1B* copies, 16 were FLU-R. The entire coding sequence (4,448 bp; 1,031,203–1,035,650 bp) of *CDR1B* has been amplified among these 30 isolates (Fig. S5). Also, increased *ARR3* (4,389 bp; 280,091–284,479 bp, including upstream ORF CPAR2_601040) copy number was detected in all cluster I isolates, plus 12 additional FLU-S isolates outside of this cluster (Fig. 1; Table S8). In contrast, while *RTA3* CNV was not observed in cluster I isolates, they were found in clusters II, III and V isolates (Fig. 1). The Kruskal–Wallis test indicated that increased *ERG11* and *CDR1B* copies were strongly and positively correlated (*P*<0.001) with Flu MICs (Fig. 2a, b), while elevated *ARR3* copies were also positively but less strongly associated with Flu MICs (*P*<0.05) (Fig. 2c). *RTA3* copy number showed negative statistical association with Flu MIC (*P*<0.05) (Fig. 2d). For the remaining four genes showing CNV (*GZF3, ERG28, LIP1* and *LIP2),* the number of isolates with increased gene copies was all small (<7 isolates) and the estimated CNVs ranged between 2 and 3 (Table S8), and their increased copy numbers did not appear to correlate with Flu resistance.

Aside from the ‘samtools depth’ module estimation approach, CNV was explored using CNVpytor v1.3.1 [58] with bin size set as 100 bp. The CNV results have been merged over the 51 samples, and low-quality calls were filtered, such as calls with more than half not uniquely mapped reads, non-confident calls (*P*≥0.0001), calls with more than 50% Ns in the reference genome, calls smaller than 1,000 bp and calls close to gaps in the reference genome (<18,000 bp). Our analyses identified several genomic regions ranging from 2,900 to 214,000 bp that might have undergone duplications and deletions (Table S9). For instance, for the isolates with high MICs to FLU in cluster I, an increased copy number of chromosome NW_023503279.1 : 868101-873500 ranging from 4 to 12 copies was observed. These regions contained some genes known to be associated with drug resistance such as *ERG11*, and their duplications may represent traits of adaptations, consistent with results from the first approach. However, the increased copy number in several other regions, e.g. NW_023503282.1:24801-46400 ranging from 3 to 6 copies, was present in both antifungal-resistant and -sensitive isolates. Their adaptive significance remains unknown.

**Fig. 3.**
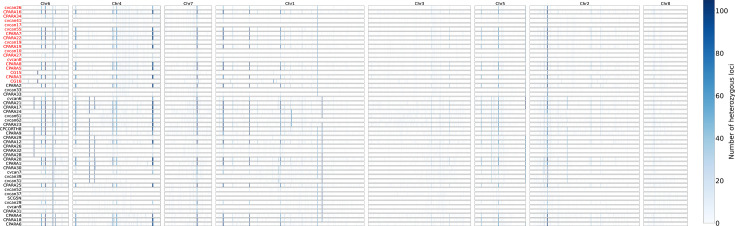
Loss of heterozygosity pattern of 51 *C. parapsilosis* strains across 8 genome scaffolds. Each block illustrates the heterozygous level for a scaffold which was divided into non-overlapping 5,000 bp windows. The intensity of the blue colour represents the number of heterozygous loci for each window.

The genome-wide heterozygosity patterns for all 51 isolates are shown in Fig. 3. The 51 isolates differed in their amounts of heterozygous loci, ranging from 2,060 to 16,575. None of the 51 isolates was completely homozygous. Among the five clusters, the average overall heterozygosity was highest in cluster V (2,429), followed by clusters III (1,970), IV (1,622), I (1,435) and II (1,402). Interestingly, while all 8 chromosomes in all 51 strains contained heterozygous loci, there were differences in the frequency of heterozygosity within and among the chromosomes. For example, chromosome NW_023503284.1 had the highest overall heterozygosity, with an average of 255 heterozygous loci present in this 957,321 bp scaffold (0.24 per kb). NW_023503280.1 and NW_023503277.1 had similarly low heterozygosity, at 0.07 per kb. Various heterozygosity frequencies were also observed among different strains for each chromosome. The heterozygous loci ranged from 16 to 117 in NW_023503277.1 across strains. Large chromosomal regions with limited or no heterozygous loci were likely the results of recent loss of heterozygosity events. Such regions are found in all isolates on all chromosomes (Fig. 3). For FLU-R isolates, while several chromosomal regions such as those highlighted on chromosomes 1 (NW_023503281) and 3 (NW_023503283) had limited heterozygosity, such low heterozygosity was also found in several FLU-sensitive isolates. Thus, the loss of heterozygosity alone cannot account for differences in fluconazole susceptibilities.

### Higher expression of *ERG11* and *CDR1b* in FLU-R *C. parapsilosis*

**Fig. 4.**
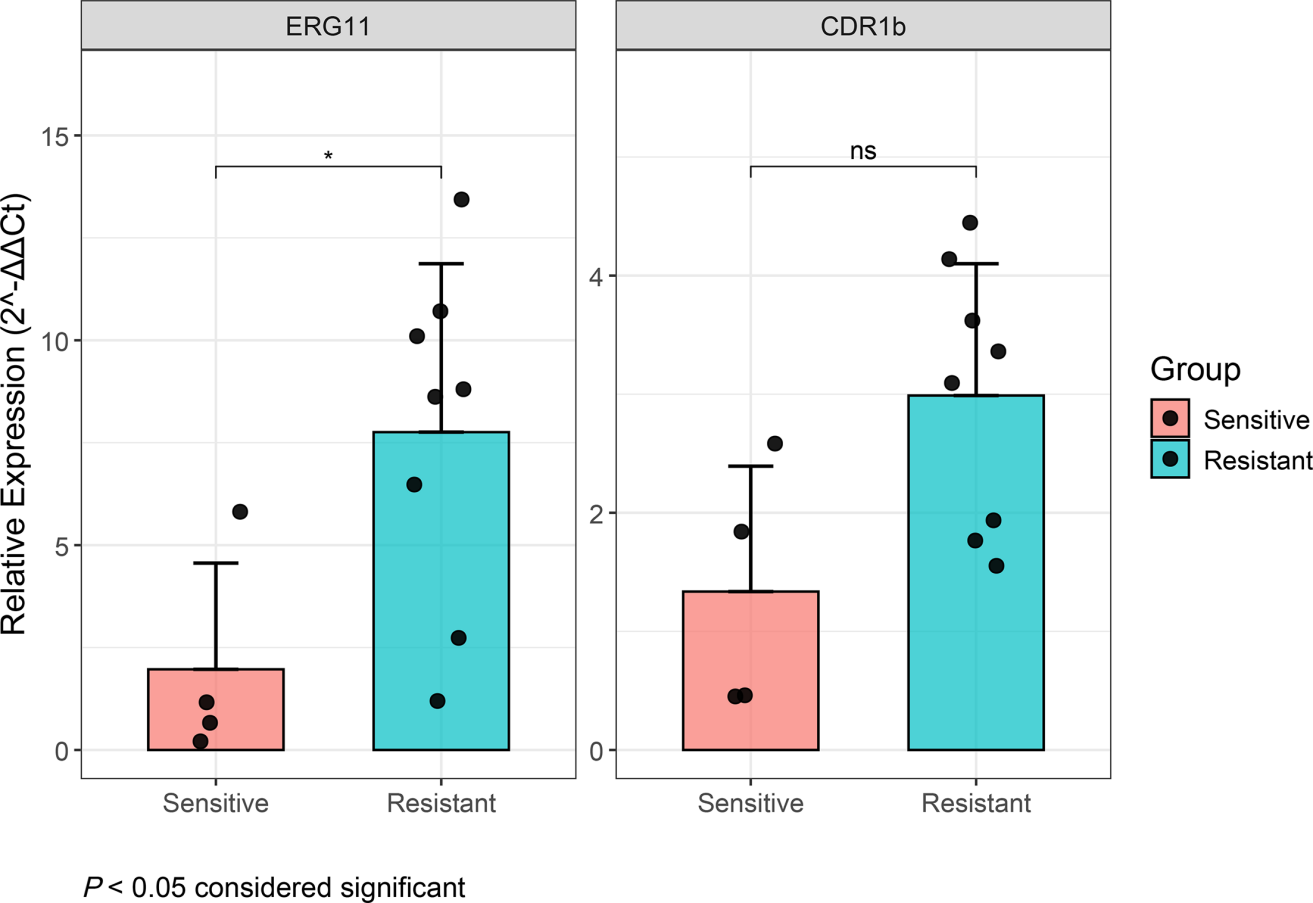
Gene expression levels of *ERG11* and *CDR1b* in representative FLU-R and FLU-S *C. parapsilosis isolates* assessed by qPCR using *ACT1* as the reference. Gene expression levels were displayed using bar charts, with individual data points overlaid as dot plots. Error bars represent the standard error of the mean for each group. Statistical analysis was performed using the Wilcoxon rank-sum test. * denotes *P*<0.05.

The expression levels of *ERG11* and *CDR1b* were compared between FLU-R (*n*=8) and FLU-S (*n*=4) isolates using RT-qPCR. Notably, *ERG11* expression was significantly higher among the FLU-R strains compared to the sensitive ones (*P*<0.05) (Fig. 4). Similarly, the expression of *CDR1b* was greater in FLU-R isolates than FLU-S isolates, but the difference was not statistically significant (*P*=0.073) (Fig. 4).

## Discussion

In this study, the proportion of clinical isolates of *C. parapsilosis* resistant to fluconazole was high. All FLU-R isolates in cluster I may have survived and persisted outside the human hosts and on inanimate surfaces for a long period of time, which may be similar to the behaviour of *C. auris*. The persistence of FLU-R isolates spanning a range of time has been reported in various countries [68,69]. For instance, FLU-R *C. parapsilosis* (FLU-R *Cp*) has been recovered over a period of 3–7 years [27]. Our result here is consistent with previous reports and suggests the ability of FLU-R *Cp* to persist long term in Qatar hospitals.

The observed rate of FLU-R *Cp* in this Qatari population (36%) is comparable to those reported in several recent studies of *C. parapsilosis* in other geographic regions [5,14, 15, 70]. For example, ~30% of *C. parapsilosis* isolates from the female reproductive tract in Hainan Province, China, were resistant to fluconazole, itraconazole and voriconazole [70]. Greater popularity of fluconazole in candidemia treatment due to lower cost compared to echinocandins could be the driving force in the elevated frequency of FLU-R *Cp* [1]. However, only 4 of the 18 patients with FLU-R *Cp* were ever treated with fluconazole (or any azole drug). Similarly, none of the patients with triazole-resistant *C. parapsilosis* reported earlier from Hainan had triazole antifungal treatment prior to sampling and diagnosis [70]. The results suggest that most triazole-resistant *C. parapsilosis* strains in Qatar and other places were not driven by triazole selection within individual patients during drug treatment but instead were acquired from environmental or other sources. Indeed, there is increasing evidence indicating triazole fungicide applications in agriculture and food industry as a major driver for the emergence and spread of triazole-resistant fungal pathogens in humans [71,72].

Ninety-six per cent of *Cp* isolates analysed here were recovered from HAIs, of which 15 were isolated from COVID-19 patients, who had stayed in COVID-19-designated ICUs during the pandemic in Qatar. Although these COVID-19-related isolates did not cluster into one clonal group but were distributed in at least three major clusters, the pattern was similar to that found for the Qatari *N. glabratus* [33], in which COVID-19-related isolates were distributed in four different genetic clusters. In contrast, COVID-19-associated *C. auris* had a higher level of clonality among Qatari isolates [34]. Smaller genetic variation among isolates from COVID-19 patients compared to related isolates in the cluster I indicated that a portion of the COVID-19-associated isolates may have come from a recent common ancestor. The pandemics could have driven the expansion of certain clones due to nosocomial transmission. Medical teams and staff caring for the patients in different wards/units may be the cause of HAIs. Also, hospital environmental niches, such as bedside carts, bed rails, infusion pumps and cardiac monitors, have been proposed as the source of FLU-R *Cp* [14]. Multiple genomic epidemiological investigations on *C. parapsilosis* have been performed in various countries, and nosocomial transmission has been reported in Canada [31] and Turkey [23]. Notably, our data indicated the isolates from different hospitals grouped together in the same phylogenetic cluster, particularly those from COVID-19 patients in clusters II and IV. This demonstrates the value of WGS analysis to detect and characterize phylogenetic clusters and outbreaks and shows the importance of monitoring local antifungal resistance trends in invasive fungal infections.

The spread and persistence of drug-resistant *C. parapsilosis* strains in clinical settings could lead to the failure of antifungal treatment. While FLU-R *Cp* was common in Qatar, all the Qatari isolates were sensitive to echinocandins, suggesting that a shift in treatment strategies from the commonly recommended to a new one is needed in Qatar. Echinocandin with amphotericin B is an alternative option for FLU-R isolates. Among oral antifungals, posaconazole, isavuconazonium sulphate and voriconazole remain potential options for FLU-R *Cp* infections [73,74]. However, the efficacy of alternative azoles in the current setting has not been well established, and most experts advocate for the use of a different antifungal class [73,74]. Therefore, prevention strategies should consider appropriate control measures against both exogenous and endogenous infection reservoirs [1].

In our study, none of the FLU-R isolates in Qatar carried the known mutations that result in reduced drug binding at the Erg11 such as Y132F and K143R [5,8, 26]. Similarly, other mutations associated with loci that confer resistance or regulate or activate the drug efflux such as Mrr1 (A854V), Tac1 (L518F, G650E), Erg3 (G111R) and Upc2 (Q371H) [8,19, 20] were also not detected. The lack of signature mutations in azole target loci and ergosterol synthesis pathway genes indicated that these isolates have developed other mechanisms to confer fluconazole resistance.

CNV represents one of the molecular mechanisms to confer fluconazole resistance. In this study, *ERG11* CNV was discovered in all 18 FLU-R isolates in cluster I, amid one fluconazole-sensitive isolate. Also, we have detected CNV in *ERG11* in four FLU-R *Cp* isolates in South Korea, which also had no reported mutation in *ERG11, TAC1* and *MRR1* [75], using the same samtools suite (data not shown). Previously, CNV in *ERG11* has been reported in *C. parapsilosis* in two studies [64,65]. Genomic analysis from a Chinese team revealed 21 azole-resistant *C. parapsilosis* isolates had either whole-chromosomal aneuploidy or chromosomal segmental duplication of 2.3–12.1 kb [65]. In contrast, a study conducted in the USA found two South African isolates exhibiting CNV among 35 FLU-R isolates; the entire ORF was amplified in the first one, while only the promoter region was amplified in the second [64]. Based on the RT-qPCR findings, the greater expression of *ERG11*, associated with greater copy numbers, could lead to increased fluconazole resistance in clinical settings. Apart from *ERG11*, we also detected CNV in *CDR1B*, *RTA3* and *ARR3*, which have been reported to be associated with increased azole MICs or other selective pressures in *C. parapsilosis* [76,79]. Specifically, increased expression of *CDR1B*, which encodes an ATP-binding cassette (ABC) transporter, has been reported in FLU-R *Cp* isolates in a previous study [64] and in this study, though not statistically significant. Although the association between Flu MICs and copy numbers of *CDR1B* as well as *RTA3* was statistically significant in our study, increased amplification of these two genes was unlikely to be sufficient to cause fluconazole resistance. This is because several FLU-S isolates also had elevated CNV at these two genes. Bergin *et al.* also argued that higher *CDR1B* copy numbers did not correlate well with higher MIC, and Nanopore sequencing data suggested that *CDR1B* (CPAR2_304370) in the original *C. parapsilosis* CDC317 genome assembly could contain two nearly identical genes in tandem. *RTA3* encodes a putative phosphatidylcholine floppase, and its increased copy number was reported to be associated with miltefosine resistance, but not fluconazole, among clinical *C. parapsilosis* isolates [77]. The increased CNV of *ARR3* did not appear to be associated with increased fluconazole resistance in the current investigation. Selmecki argued that the increased amplification of these two genes plays important roles in physiological adaptation in *C. parapsilosis* [78].

CNV in *ERG11* has been implicated as a resistance mechanism to fluconazole in various *Candida* species [8], such as *C. tropicalis* [66], and other fungal species like *Malassezia pachydermatis* [80] and *Trichophyton indotineae* (as homologues of CYP51) [81]. Indeed, CNV is one of the major molecular mechanisms linked to antifungal resistance and adaptations. CNV and segmental aneuploidy in *C. albicans* have been associated with decreased susceptibility or increased tolerance and resistance to antifungal drugs in clinical isolates and in laboratory evolution experiments [82,84].

GWAS detected several putative variants that could be associated with resistance to Flu and Fc. These variants are in genes of unknown functions at present. Specifically, one missense and a silent mutation in *ALS3* (one of the 16 members in the gene family) could be linked to resistance to Fc and Flu, respectively. Cell surface Als (agglutinin-like sequence) proteins can activate cell–cell adhesion that helps micro-organisms to bind to host cells and regulate the adhesion of biofilms [85]. Measuring the level of *ALS3* expression may be required to elucidate its role on antifungal resistance.

Efg1 and Ume6 are regulators of filamentation, morphogenesis, biofilm formation and white-opaque switching processes in *Candida* [62,86,89]. In *C. parapsilosis,* Efg1 regulates biofilm formation and a phenotypic switch [61,62]. Elevated azole resistance conferred by *C. parapsilosis* in biofilms has been considered a growing clinical problem [1,90, 91]. All strains of cluster I possessed unique mutations in *EFG1* and *UME6* as compared to samples of other clusters and unrelated isolates. It is possible that *C. parapsilosis* isolates with greater biofilm formation ability could be more likely to spread across the hospital environment. However, it is also possible that these genetic variabilities may represent natural neutral polymorphisms with no or limited functional implications.

CNV and loss of heterozygsity (LOH) events may involve large numbers of genes, and the co-occurrence or stepwise acquisition of these mechanisms may result in azole resistance in yeast adaptation [84]. Azole-resistant strains of *C. parapsilosis* appeared to exhibit reduced genetic diversity, LOH, and were enriched in a single genetic cluster in our Qatari sample of *C. parapsilosis*. Through LOH, drug-susceptible alleles may be lost and while those conferring drug resistance could be doubled through gene conversion such as mitotic crossing over to replace the drug-susceptible alleles. The doubling of drug-resistant alleles could help elevate MIC and, along with a further increase in copy numbers through whole or partial chromosomal duplications, may result in strains with very high MIC. Such phenomena have been observed in *C. albicans* and *Pichia kudriavzevii* (formerly *Candida krusei*) [82,83, 92].

There are several limitations to this study. First, we were unable to perform a molecular genetic experiment to verify the role of CNV detected in conferring resistance to a susceptible isolate. Second, the sample size (*n*=51) was not large; however, the data contributed to the understanding of *C. parapsilosis* in the Middle East region. Third, we have MIC data based on *in vitro* measurement in more than one platform. It is not clear whether this may have translated to treatment failure in clinical settings. Despite these shortcomings, our study revealed the presence, persistence and potential transmission of FLU-R *Cp* in Qatari hospital settings. Our findings extended and supported the data of Bergin *et al.* that increased copy numbers of *ERG11* and *CDR1B* could result in increased fluconazole resistance [64]. Additional screening of *C. parapsilosis* samples from healthcare workers, indwelling devices and environmental surfaces within clinical environments will improve understanding of their nosocomial transmission pattern and help prevent the spread of FLU-R *Cp* [93]. In addition, extensive environmental sampling such as fruits, especially stored ones, that are known to harbour *C. parapsilosis* should be conducted to investigate the potential source(s) of the FLU-R *Cp* strains in Qatar [72,94].

## Supplementary material

10.1099/mgen.0.001653 Uncited Supplementary Material 1.

10.1099/mgen.0.001653 Uncited Supplementary Material 2.

## Abbreviations

BLINK: Bayesian-information and Linkage-disequilibrium Iteratively Nested Keyway
CLSI: Clinical and Laboratory Standards Institutes
CNV: copy number variation
COVID-19: coronavirus disease 2019
CSF: cerebrospinal fluid
ECVs: epidemiological cutoff values
FarmCPU: Fixed and random model Circulating Probability Unification
FLU-R: fluconazole-resistant
FLU-R *Cp*: FLU-R *Candida parapsilosis*
GWAS: genome-wide association study
HAI: hospital-acquired infection
HGH: Hamad General Hospital
HMGH: Hazm Mebaireek Hospital
ICUs: intensive care units
LD: linkage disequilibrium
MIC: minimum inhibitory concentration
NHSN: National Healthcare Safety Network
QTNs: quantitative trait nucleotides
REM: random effect model
SDD: susceptible dose-dependent
SM: Sidra Medicine
TD: Tajima’s D
WGS: whole-genome sequencing
