# Supplementary material for: Persistence and spread of fluconazole-resistant Candida parapsilosis clinical isolates associated with increased ERG11 copies in Qatar
Source: Microb Genom. 2026 Feb 20;12(2):001653. doi: 10.1099/mgen.0.001653 (PMC12927641; doi:10.1099/mgen.0.001653)
Supplement: Uncited Supplementary Material 1. [file mgen-12-01653-s001.pdf]

Supplementary materials

Supplementary text – RT-qPCR evaluation of *ERG11* and *CDR1b* expression in FLU-R and FLU-S *Candida parapsilosis* Isolates

Table 1 Primer sequences and PCR product sizes for ACT1, CDR1b, and ERG11.

Gene	Primer name	Sequence (5'->3')	Aplicon size	Reference
Actin	ACT1-F	AGAATCGATTTGGCTGGTAG	121 bp	Daneshnia F et al. 2022
	ACT1-R	CACAATTTCTCCTGATGTCTC		
CDR1b	CDR1b-F	CCACTTGTGTGCGTTGTAA	93 bp	Doorley et al. 2022
	CDR1b-R	TGTTCCGCAGTAGGGTCT		
ERG11	ERG11-F	GTTGGTCAGCCGTATCTT	110 bp	Daneshnia F et al. 2022
	ERG11-R	CACCGTCATTACTCTACCCA		

qPCR reaction and thermal cycling conditions

Each 20 µl reaction contained:

- 10 µl of SYBR Green Master Mix
- 0.5 µl of forward primer
- 0.5 µl of reverse primer
- 2 µl cDNA
- 7 µl of nuclease free water

The qPCR was performed on a Quant Studio 6 Flex Real-Time PCR System (Thermo Fisher) using the following thermal cycling conditions: Initial Denaturation: 95 ºC for 10 min, followed by 40 cycles of amplification: denaturation at 95 ºC for 15 s, annealing at 55 ºC for 30s and extension at 72 ºC for 1minute. The specificity of the PCR amplification was confirmed by performing a melt curve analysis at the end of the run, as well as by running the PCR products on a 2% agarose gel to confirm the expected amplicon size and absence of non-specific bands.

References

Daneshnia F, Hilmioğlu Polat S, Ilkit M, Shor E, de Almeida Júnior JN, Favarello LM, Colombo AL, Arastehfar A, Perlin DS, 2022. Determinants of fluconazole resistance and the efficacy of fluconazole and milbemycin oxim combination against *Candida parapsilosis* clinical isolates from Brazil and Turkey. Frontiers in fungal biology, 3, 906681.

Doorley LA,Rybak JM, Berkow EL,Zhang Q, Morschhäuser J, Rogers PD, 2022. Candida parapsilosis Mdr1B and Cdr1B Are Drivers of Mrr1-Mediated Clinical Fluconazole Resistance. Antimicrob Agents Chemother 66:e00289-22.

Legends

Figure S1. Boxplots and statistical tests showing the variation between pairwise genetic distance (including heterozygous sites) between groups of samples (a) COVID-19 vs non-COVID-19 vs all samples (Wilcoxon rank-sum test (non parametric) with Bonferroni correction), (b) Clade I vs non-Clade I vs all samples (Wilcoxon rank-sum test (non parametric) with Bonferroni correction), (c) COVID-19 vs non-COVID-19 within clade I (Cohen's d)

Figure S2. Genomic comparison between isolates from Qatar and other countries based on whole-genome SNPs. Genetic relationships among these 51 Qatari isolates and the 228 strains from other countries were inferred based on SNP calls using CDC317 as the reference genome.

Figure S3. Genetic relationships among 51 *C. parapsilosis* isolates overlaid with the missense variants and stop codons gained detected from the 53 panel loci. The location and annotation of these variants was listed in Table S10.

Figure S4. QQ plots and Manhattan plots for (a) Flu, and (b) Fc from GWAS. On the left, the y-axis of the QQ plot represents the quantiles of the observed p-values, while the x-axis represents the quantiles of the expected p-values under the null hypothesis. The red diagonal reference line represents the expected distribution of data. The blue circles deviating from the diagonal at the tail indicate that certain genetic variants are associated with the drug susceptibility. On the right, each SNP was represented by a dot on the Manhattan plots. x-axis shows the chromosomal positions of the markers. The y-axis represents -log10(p-value) for each SNP. The horizontal lines indicated the significance threshold to identify statistically significant associations for each analysis. Gene ID with significant SNPs was labelled on the Manhattan plots.

Figure S5. Screenshots from Integrated Genomic Viewer (IGV) showing the increased read depth (coverage) for isolates CPARA5. (a) the increased copies/ amplification of the entire *ERG11* and upstream and downstream loci. (b) the increased copies of the entire *CDR1B*

Fig S1

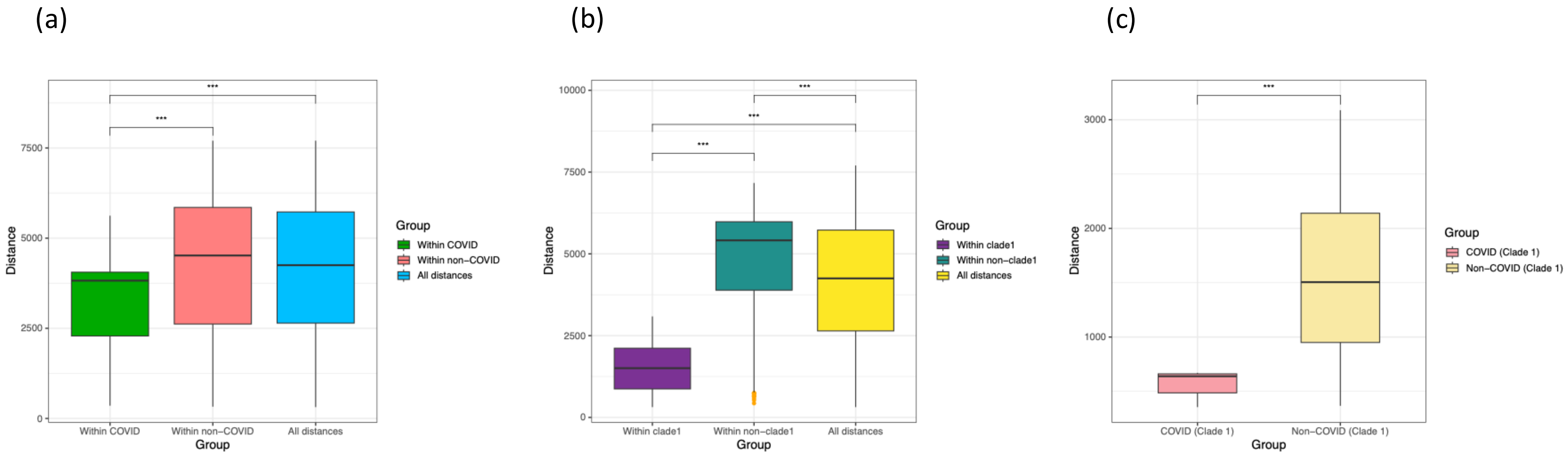
(a)
(b)
(c)
***
Distance
Group
Within COVID
Within non−COVID
All distances
Within clade1
Within non−clade1
COVID (Clade 1)
Non−COVID (Clade 1)
0
1000
2000
2500
3000
5000
7500
10000

Fig. S2

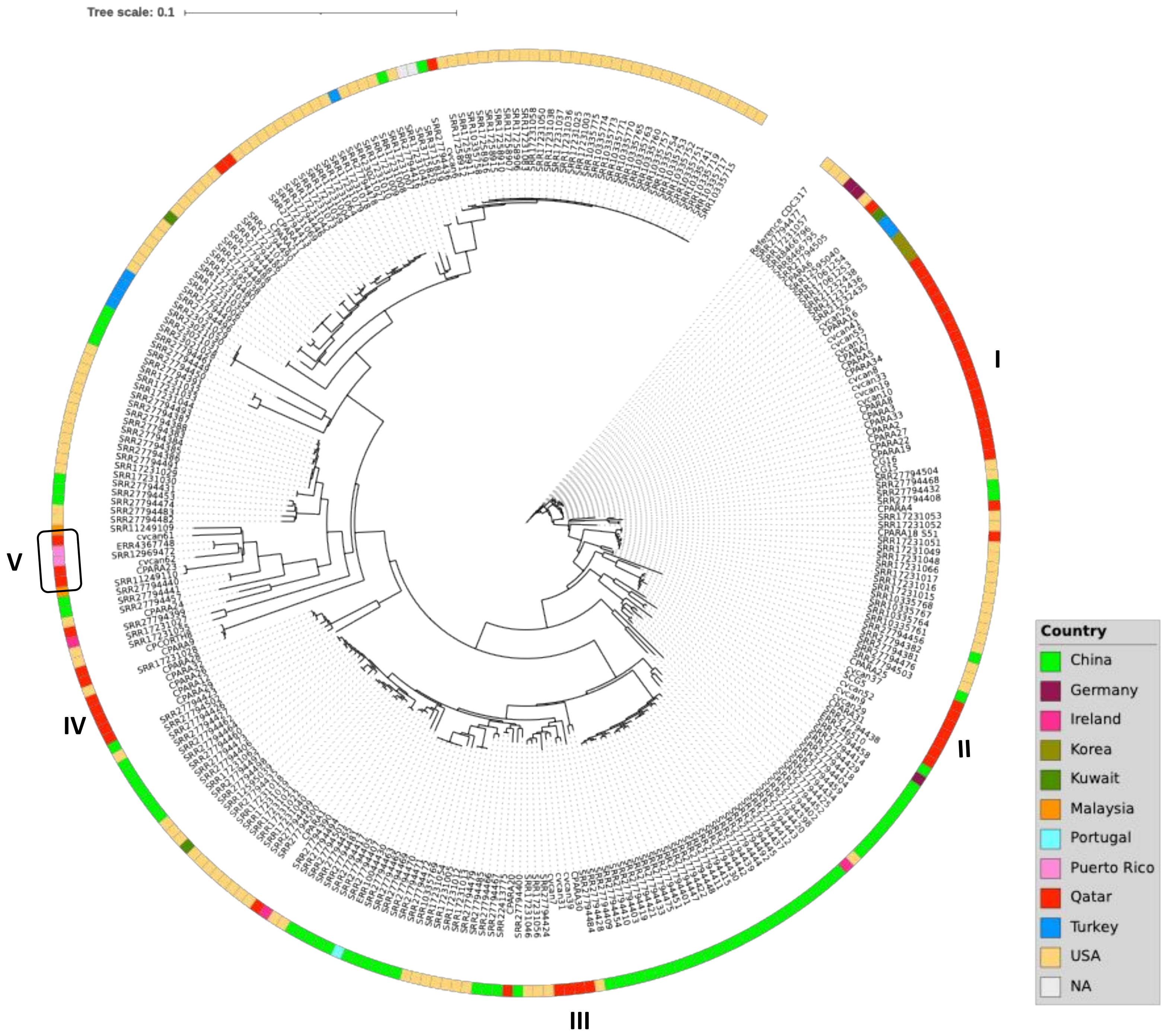
Tree scale: 0.1
I
II
III
IV
V
Country
China
Germany
Ireland
Korea
Kuwait
Malaysia
Portugal
Puerto Rico
Qatar
Turkey
USA
NA

Fig. S3

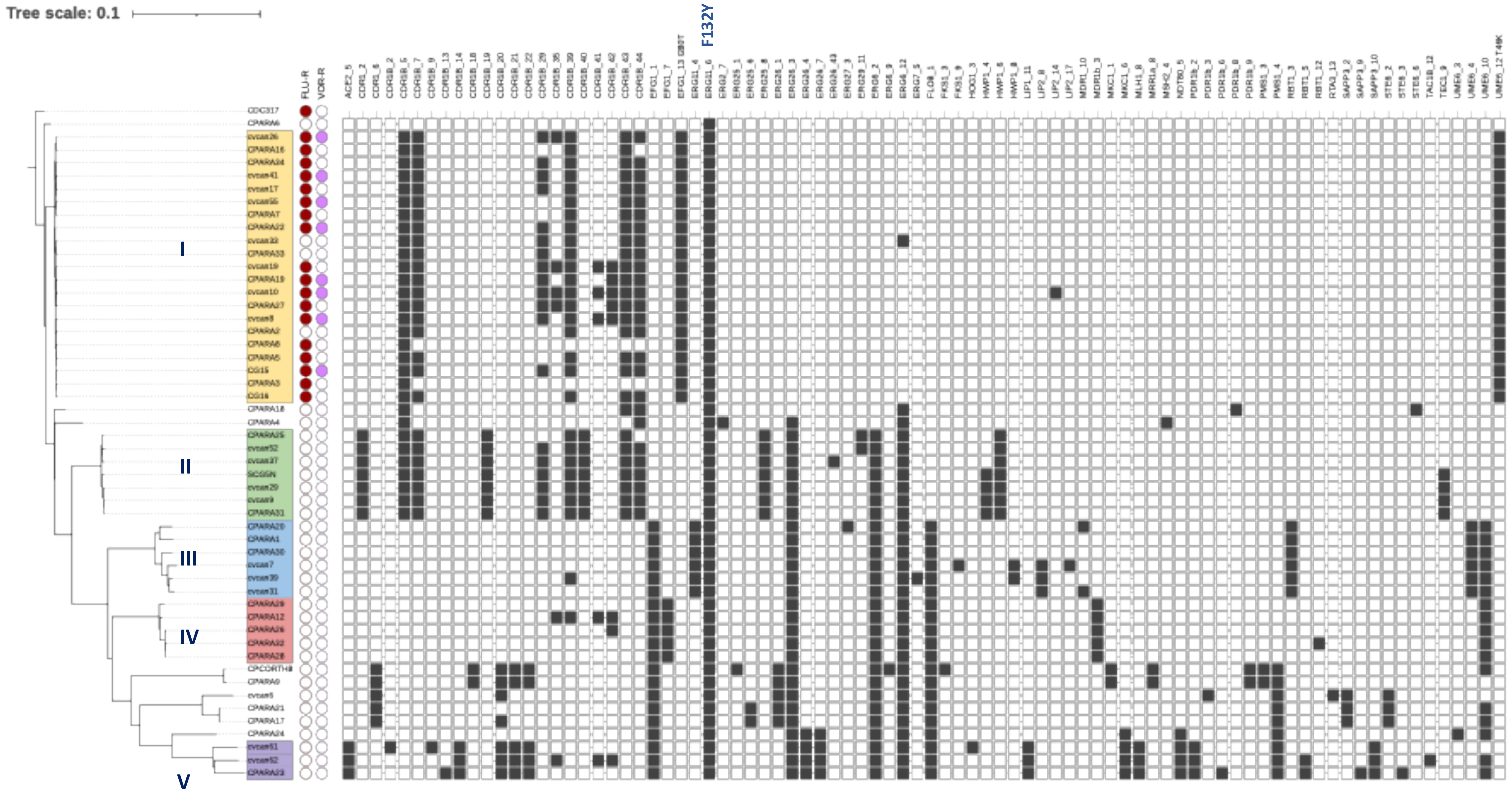

Fig. S4

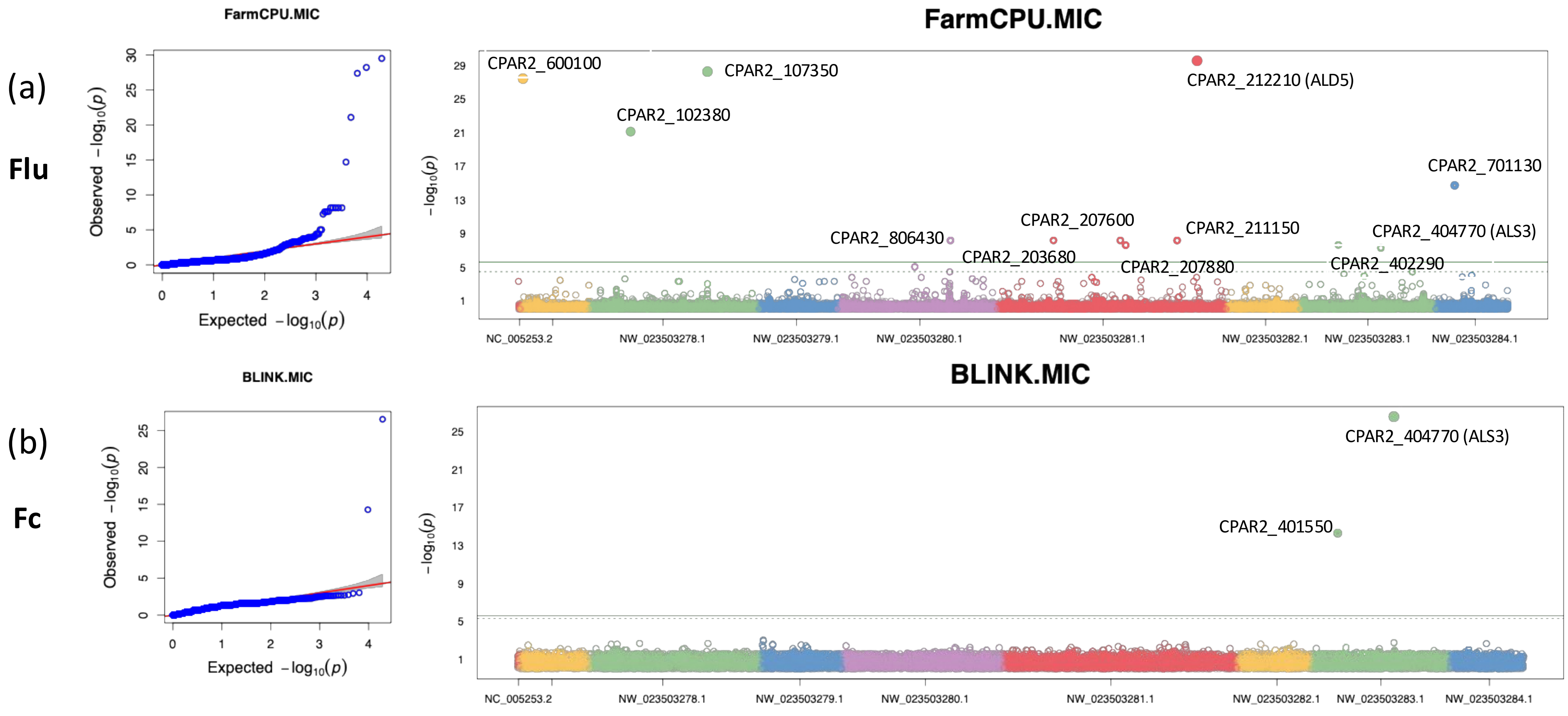
(a)
Flu
FarmCPU.MIC
Observed −log10(p)
Expected −log10(p)
FarmCPU.MIC
CPAR2_600100
CPAR2_107350
CPAR2_212210 (ALD5)
CPAR2_102380
CPAR2_701130
CPAR2_207600
CPAR2_211150
CPAR2_806430
CPAR2_404770 (ALS3)
CPAR2_203680
CPAR2_402290
CPAR2_207880
−log10(p)
NC_005253.2
NW_023503278.1
NW_023503279.1
NW_023503280.1
NW_023503281.1
NW_023503282.1
NW_023503283.1
NW_023503284.1
(b)
Fc
BLINK.MIC
Observed −log10(p)
Expected −log10(p)
BLINK.MIC
CPAR2_404770 (ALS3)
CPAR2_401550
−log10(p)
NC_005253.2
NW_023503278.1
NW_023503279.1
NW_023503280.1
NW_023503281.1
NW_023503282.1
NW_023503283.1
NW_023503284.1

Fig. S5

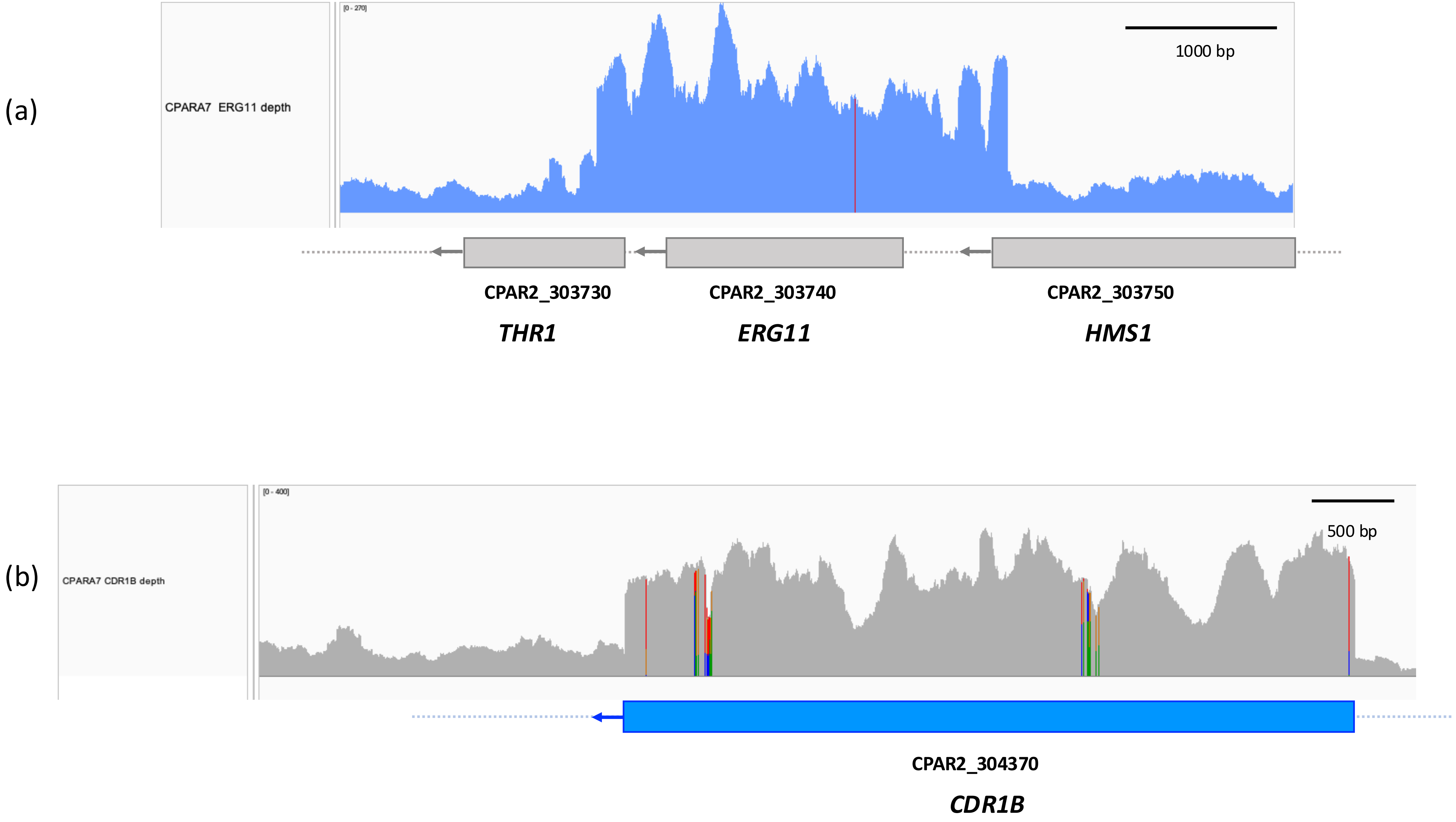
(a)
CPARA7 ERG11 depth
[0 - 270]
1000 bp
CPAR2_303730
CPAR2_303740
CPAR2_303750
THR1
ERG11
HMS1
(b)
CPARA7 CDR1B depth
[0 - 400]
500 bp
CPAR2_304370
CDR1B
